# Training Effectiveness of The Inertial Training and Measurement System

**DOI:** 10.2478/hukin-2014-0107

**Published:** 2014-12-30

**Authors:** Mariusz Naczk, Wioletta Brzenczek-Owczarzak, Jarosław Arlet, Alicja Naczk, Zdzisław Adach

**Affiliations:** 1 Department of Physiology, Faculty of Physical Culture, Gorzow Wielkopolski, Poland.

**Keywords:** strength, power, ITMS training, shoulder muscles

## Abstract

The purpose of this study was to evaluate the efficacy of inertial training with different external loads using a new original device - the Inertial Training and Measurement System (ITMS). Forty-six physical education male students were tested. The participants were randomly divided into three training groups and a control group (C group). The training groups performed inertial training with three different loads three times weekly for four weeks. The T0 group used only the mass of the ITMS flywheel (19.4 kg), the T5 and T10 groups had an additional 5 and 10 kg on the flywheel, respectively. Each training session included three exercise sets involving the shoulder joint adductors. Before and after training, the maximal torque and power were measured on an isokinetic dynamometer during adduction of the shoulder joint. Simultaneously, the electromyography activity of the pectoralis major muscle was recorded. Results of the study indicate that ITMS training induced a significant increase in maximal muscle torque in the T0, T5, T10 groups (15.5%, 13.0%, and 14.0%, respectively). Moreover, ITMS training caused a significant increase in power in the T0, T5, T10 groups (16.6%, 19.5%, and 14.5%, respectively). The percentage changes in torque and power did not significantly differ between training groups. Electromyography activity of the pectoralis major muscle increased only in the T0 group after four weeks of training. Using the ITMS device in specific workouts allowed for an increase of shoulder joint adductors torque and power in physical education students.

## Introduction

The role of muscle strength during everyday activities, sports, and manual labor is widely understood. Strength, along with the velocity of movements, determines the level of power. A key consideration for the long-term development of an athlete’s maximal power is the integration of numerous power training techniques (Cormie et al., 2011). Inertial training can complement or be an alternative to other training methods. Inertial training is performed with a specific device that utilizes inertial resistance, which differs from more traditional resistance modalities. During concentric contraction the load (for example flywheel) is accelerated, while during eccentric contraction the load is decelerated by the same muscle group which worked during the concentric contraction. It is known that during inertial training the eccentric phase is stronger and muscle activation is greater in comparison to standard weight training (Norrbrand et al., 2008; 2010; Onambele et al., 2008). Moreover, numerous studies have proven that eccentric contractions elicit greater muscle hypertrophy than concentric ones (Bamman et al., 2001; Moore et al., 2005; Roig et al., 2009). Thus, a strong eccentric phase, which occurs during inertial training (greater than in standard weight training) can elicit a great muscle strength increase. According to available literature, several weeks of inertial training may cause a significant increase of the maximal torque in the biceps brachii muscle (Albert et al., 1994) or can improve knee extensor power (Onambele et al., 2008). However, McLoda et al. (2003) found that ball velocity, arm velocity, and throwing accuracy were not affected by inertial training in baseball and softball players. In the aforementioned studies, two inertial devices were used: the Impulse Training System and the YoYo ergometer. These devices are user - friendly but due to their construction either the range of motion is limited or determination of specific movement mode is impossible. In this situation we decided to design and construct the Inertial Training and Measurement System (ITMS), which uses a different technology than other devices (see Methods section). In our opinion the ITMS combines advantages of both mentioned above devices. The ITMS is a new, universal device, which enables regulation of training loads by increasing the speed of movement or resistance, depending on the training objectives (the improvement of muscle power vs. strength). Moreover, the ITMS also allows for exercises to be performed throughout a range of motion at various speeds. The ITMS makes it possible to establish a lot of movement modes, involving various muscle groups, and allows specific movements, which are typical of various sport disciplines or professional activities, to be performed. Since our device combines the advantages mentioned above, it is a novelty in inertial training and can be an alternative to other existing inertial devices. The advantages of the ITMS indicate it can be very useful in practice, but the training protocols and its efficacy are not known yet.

The purpose of this pilot study was to evaluate the efficacy of inertial training with different external loads: 0 kg, 5kg, 10 kg using the Inertial Training and Measurement System. For evaluating training efficacy using the ITMS device, shoulder joint adductors training was performed.

## Material and Methods

### Participants

Forty-six physical education male students were included in the study. The participants were randomly divided into three training groups and a control group (C group). All subjects from the first group (the T0 group) participated in training with no load in addition to the mass of the flywheel (19.4 kg); while, the flywheel was loaded with additional 5 kg for all subjects from the second group (the T5 group) and with additional 10 kg for all subjects from the third group (the T10 group). The somatic characteristics of the participants are summarized in Table 1. The physical activity of participants was high. Before and during the study, they followed basketball (90 min per week), swimming (90 min per week), gymnastic (45 min per week), and athletic (45 min a week) training. The total exercise time per week was 270 min. None of the subjects was a competitive athlete. All participants were required to maintain their regular daily activities during the training period. All students were informed about the procedures, risks, and benefits and signed an informed consent form. All procedures were approved by the Commission of Bioethics at the Poznan Medical University, with approval based on the Declaration of Helsinki.

### Training

Training was performed with a new developed device - the Inertial Training and Measurement System (ITMS), which was designed and constructed by an inter-university group from the Faculty of Physical Culture in Gorzow Wielkopolski (department of the University School of Physical Education in Poznan) and the Faculty of Mechanics University of Zielona Gora (Picture 1). This device is comprised of a steel frame attached to the ground with an inertial wheel (flywheel) placed inside. The radius of the flywheel is 506 mm. A rope is mounted on the circumference of the wheel. Prior to exercise, subjects were seated on a bench and the length of the rope was adjusted to the distance between the device and the bench. In the “0” position, the rope was fully extended and tense. To begin exercising, subjects pulled the rope by adducting their arm and moving the flywheel (the wheel rotated approximately 90 degrees).

**Figure 1:**
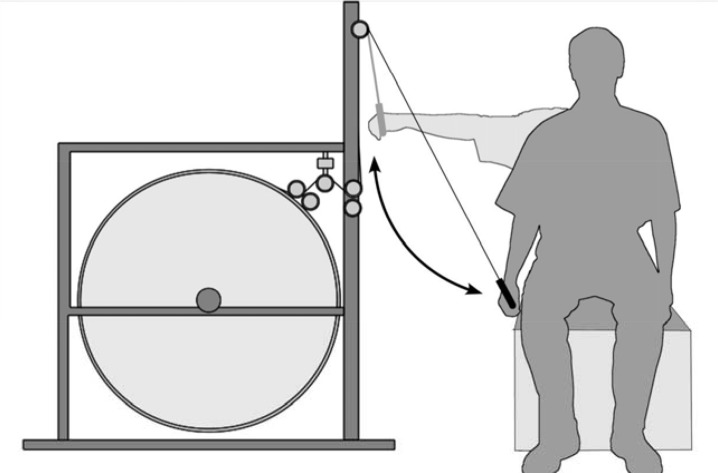
Participants position during training and the ITMS

Inertial training was performed three times per week for a period of four weeks (every Monday, Wednesday, and Friday, between 9 am – 12 pm). Training was carried out by the same two researchers. Each training session included three exercise sets involving the shoulder joint muscles. One set included abduction and adduction of the right and left arms (without rest) at the shoulder joint by a participant positioned laterally to the device. The shoulder adductor muscles worked concentrically during adduction movement (the ITMS flywheel was accelerated during this phase) and eccentrically during abduction. Participants tried to adduct their arm throughout the exercise, and shoulder joint abduction was only forced by the flywheel mass of inertia. In the starting position, the arm was abducted from the trunk approximately 90 degrees (to the shoulder level). The subject positioning during ITMS training is shown in Figure 1.

The participants exercised with different loads (and following it different speed of movement). The T0 group used only the mass of the ITMS flywheel (19.4 kg), the T5 and T10 group had an additional load of 5 kg and 10 kg, respectively, on the flywheel. External loads and the speed of movement during training were different in each training group, all other training variables (number of sets, set duration, rest period) were the same. All subjects were asked to exercise at maximal speed, irrespective of the load. The work time of one limb amounted to 20 s per set, with a total work time for one arm of 60 s per training session (three sets). Subjects had a 2 min rest period between consecutive sets. Throughout the study period, the number of repetitions per set was progressively increased over the weeks of training in intervention groups: on average in the first set of training, from 29 to 34 repetitions in T0, from 26 to 31 repetitions in T5, and from 24 to 28 repetitions in T10. Different training repetitions for tested groups resulted from different inertial mass of the wheel. Greater inertial mass of the wheel caused slower speed of movement during exercise. Therefore, total volume of training was progressively increased during the training period, but was always similar in T0, T5 and T10 (lack of significant differences between work performed in training groups). Each training session was preceded by a standard 3 min warm up involving the upper parts of the body (synchronized arm rotations, alternating arm swings, lateral arm swings with trunk rotation, and a few slow cycles with the ITMS).

### Measures

To test the hypotheses, the maximal torque and power were measured before and after the training period. The measurements were performed during isokinetic muscle actions using a specialized Biodex 4 Pro device (Shirley, New York, USA). Data collection was preceded by a familiarization session. Biomechanical measurements were taken in a seated position (one hand grasped the device handle while the other was placed on the abdomen) as subjects abducted and adducted the right and left arm at the shoulder joint. To minimize the activity of undesired muscle groups, the participant’s trunk was stabilized using belts placed across the chest. Prior to the measurements, participants were given verbal instructions on the experiment’s design. Each isokinetic test began with two trial cycles (each comprised of shoulder joint adduction and abduction), followed by five repetitions (involving maximal strength) at an angular velocity value of π rad · s^−1^, and ranged from 10 to 90 degrees of motion (where 0 degrees corresponded to complete adduction of the arm to the trunk). Each test session was preceded by a warm up of upper limbs. Only data recorded during adduction were subject to further analyses since the inertial exercise performed by the participants involved mostly the muscles that adducted the upper limb at the shoulder joint. For every participant, the pre- and post-training biomechanical measurements took place at the same time of day (between 9 am – 12 pm). The average values of maximal torque and power from the left and right arms were used for further analysis.

Along with biomechanical measurements, the electrical activity of the pectoralis major was registered in accordance with the SENIAM standard of measurements. EMG raw signals were detected using three surface electrodes (Ag/AgCl, Skintact, Austria). Before fixing the electrodes, the skin surface was cleaned and depilated if needed. Three surface electrodes, each 15 mm in diameter - which included one ground electrode - were placed over the central part of the pectoralis major muscle (on the side of the dominant upper limb), parallel to the direction of the muscle fibers, 1 cm distance between electrodes was maintained. During the first EMG measurement (pre-training) electrode placement was marked using a non-toxic pen marker – thanks to that during the second measurement (post-training) electrodes were placed in the same place as during pre-training measurements. Electrodes were connected to a 14-bit AD converter (ME6000 Biomonitor, Mega Electronics, Finland) by cables (Mega Electronics). Registered data were low pass filtered (8–500 Hz) and sampled at 2000 Hz before being stored in a memory card with a four-channel portable Biomonitor ME6000 system (Mega Electronics, Finland). EMG data analysis was made using Mega Electronics software (MegaWin V2.21). Only the active parts of the EMG signal were analyzed based on the amplitude of EMG, median frequency, and mean power frequency using an average spectrum (MegaWin V2.21).

#### Statistical analysis

Normality of data distribution was checked using the Shapiro–Wilk test. Descriptive statistics including means and standard deviations were calculated. Analyses of variance (one-way ANOVA) were conducted to test the differences between the 4 groups (T0, T5, T10, C) in the beginning of the experiment. To perform a direct comparison between groups, a percentage change from baseline for individual subjects was calculated for each parameter using the formula:

$$\mathrm{RC}[\%]=\frac{\mathrm{xpost}-\mathrm{xpre}}{\mathrm{xpre}}$$

RC−relative change, x−value measured before (xpre) and after(xpost) training

Differences in percentage changes between groups were tested with one-way ANOVA. If differences were detected, the Scheffe post hoc procedure was used to determine where the differences had occurred. Paired t-tests were used to test for significant changes within groups from pre to post training. The level of significance was set at p ≤ 0.05. Moreover, magnitude of training effects between groups was estimated with Cohen’s effect size (ES) (Cohen, 1988). ES is defined as the difference between experimental group posttest mean and control group posttest mean divided by control group pretest SD.

## Results

**Figure 2:**
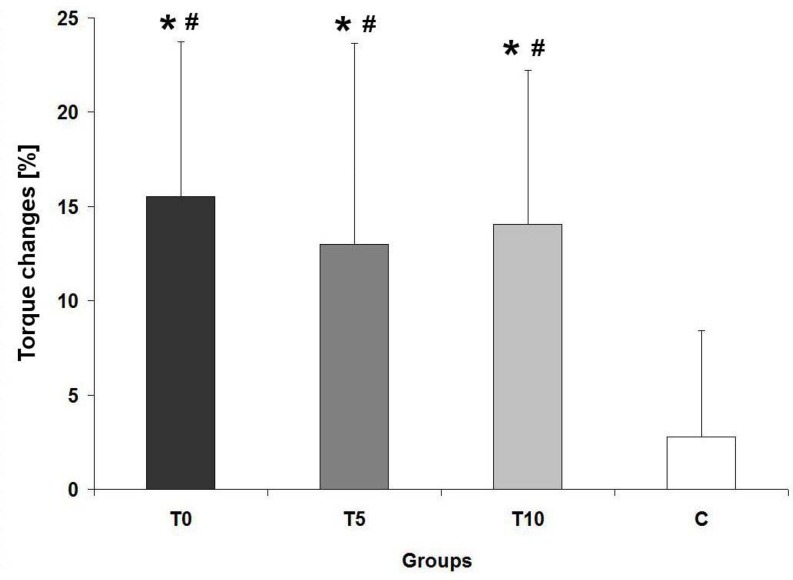
Relative changes in maximal torque T0 - group participated in training with no load in addition to the weight of the flywheel; T5 - group participated in training with the flywheel loaded with additional 5 kg; T10 - group participated in training with the flywheel loaded with additional 10 kg; C - control group; * - significant difference from baseline, # - significant difference from the control, (p ≤ 0,05).

**Figure 3:**
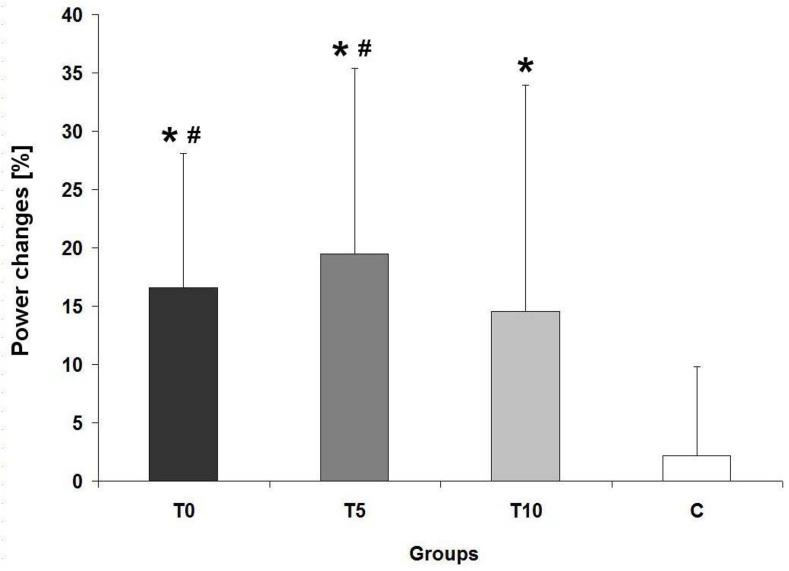
Relative changes in maximal power T0 - group participated in training with no load in addition to the weight of the flywheel; T5 - group participated in training with the flywheel loaded with additional 5 kg; T10 - group participated in training with the flywheel loaded with additional 10 kg; C - control group; * - significant difference from baseline, # - significant difference from the control, (p ≤ 0,05).

The one-way ANOVA with the pretraining values of strength and power revealed no significant differences among the 4 groups. The paired t-tests revealed that the T0, T5, T10 groups improved significantly torque and power from pretest to posttest; there was no significant changes in the control group. A relative increase of torque in the T0 group was significantly greater compared with the C group. Moreover, ES was equal 2.03. Percentage increases of torque in the T5 and T10 groups were also significantly greater than in the C group (Figure 2); effect sizes in the T5 and T10 groups equaled 0.84 and 1.42, respectively,

For power, a relative increase in the T0 group for was significantly greater compared with the C group; ES = 1.65. Percentage increases in power in the T5 also differed significantly when compared with the C group (Figure 3); effect sizes in the T5 and T10 equaled 0.92 and 0.78, respectively. Percentage changes in torque and power did not significantly differ between training groups.

EMG activity of the pectoralis major muscle in the T0 group increased following training. Median frequency, and mean power frequency in the T0 group increased significantly after training (27% and 19%, respectively); however, no significant changes in the amplitude of EMG were observed. Moreover, the percentage increase in median frequency, and mean power frequency in the T0 group was greater than in the T10 and C groups. No significant differences in EMG parameters were noted in the T5, T10, and C groups (p > 0.05 before and after the training period).

## Discussion

The results of this study demonstrated high efficacy of ITMS training in strength improvement, irrespective of external loads and speed of movement during training. The scale presented by Cohen (1988) indicates that ES < 0.41 represents a small effect, 0.41–0.70 a moderate effect, and > 0.70 a large effect. Therefore, based on the magnitudes of the effect sizes, we concluded that the ITMS training caused large increases in torque, in each training group. It is noteworthy that the relative changes in torque were similar in the T0, T5, and T10 groups (from +13.0 to +15.5%). Albert et al. (1994) noted greater discrepancy in the maximal torque improvement (4.9–26.9%) in youth participants after five weeks of inertial training of the biceps brachii (using the Impulse Training System). The percentage increases in torque observed in our training groups were slightly greater than the changes noted by Tesch et al. (2004) in the concentric and eccentric maximal voluntary contraction (MVC) of the knee joint extensors following five weeks of training with a YoYo flywheel ergometer (+11.5%). However, Seynnes et al. (2007) reported a much greater increase (38.9%) in the MVC of the knee extensors following five weeks of training on YoYo ergometers in youth participants. In contrast, Norrbrand et al. (2008) observed no significant changes in concentric and eccentric knee extension peak force after 5 weeks of inertial training in middle-aged men.

The results of this study also suggest that ITMS training is highly effective in power improvement, regardless of the external load used during workouts. In our study power improvement ranged from 15.5% – 19.5%. Greater power improvement was noted by Onambele et al. (2008) who showed a 28% increase of dynamic knee extension power in elderly women after training using a YoYo ergometer. However, the training period in this study was twice as long as in our study. Norrbrand et al. (2008) did not note significant changes in concentric (+8.9%) and eccentric (+12.0%) peak power after inertial training with a YoYo device. The results of our study suggest that inertial training using the ITMS is highly effective. We suppose that measurement conditions that were different than the training conditions (isokinetic vs. inertial) could also influence the magnitude of the effect sizes. Probably even greater improvements would be observed under specific inertial testing conditions, unfortunately measurements using the ITMS were not possible at this time.

It is interesting that different loads used by T0, T5, and T10 during training did not cause different changes in torque and power between training groups. It is possible that the loads used during training were not significantly different. However, the similar improvements in the abovementioned parameters do not mean that the induced physiological changes were also similar in trained groups. A significantly greater improvement in median frequency and mean power frequency in the T0 group in comparison to the T10 group may indicate that the physiological adaptation to ITMS training depends on the load and movement speed during training. Smaller loads may elicit neuromuscular changes followed by a muscle strength increase, while greater loads mainly cause a muscle mass increase followed by a muscle strength increase, but further research is needed to examine this issue. We did not measure muscle mass for technical reasons, however, increases in the cross-sectional muscle area can occur after only three weeks of inertial training (Seynnes et al., 2007). Results of this study indicate that improvement in neuromuscular coordination was observed only in the T0 group. Increasing of median frequency, and mean power frequency in this training group can result from improved motor unit firing rates. Our findings are consistent with those of Seynnes et al. (2007) who showed significant changes in bioelectrical muscle activity as early as the third week of inertial training. However, in the T5 and T10 groups, the EMG parameters did not differ significantly following ITMS training. Our findings in the T5 and T10 groups are in agreement with the results presented by Tesch et al. (2004), who did not observe any changes in EMG signaling after five-weeks of inertial training.

With both the plyometric and inertial methods, improvements in strength and power result from repetitive cycles of muscle shortening and lengthening (concentric contractions followed by eccentric contractions). During concentric contractions, elastic energy, previously accumulated in elastic components (such as tendons) during muscle lengthening, is utilized. The release of this energy and subsequent stimulation of muscle spindles during the eccentric phase promote increases in strength that occur during the concentric action (Asmussen and Bonde-Petersen, 1974; Chapman et al., 1985; Komi and Bosco, 1978; Koutedakis, 1989). The rate of the strength increase, however, depends on the amortization phase - the shorter the time, the more energy that can be utilized during the concentric contraction (Bosco et al., 1981). An increase in strength and power in our study could also result from an increase in the excitability threshold of the Golgi tendon organs (McNeely and Sandler, 2006). A favorable effect of the stretch-shortening cycle exercise is the stiffness of tendon structures (Kubo et al., 1999). In inertial training, tendon stiffness can increase about 136% (Onambele et al., 2008), which can impact muscle strength and power. It is possible that following the ITMS training, tendon stiffness also increased, yet it was not evaluated.

In the presented, pilot study several limitations occurred. We could not control the ITMS training parameters (duration of movement cycle, muscle force achieved during each phase of movement, etc.). It will be possible in future research due to a special computer software that was created after completion of this study. This software will also allow to evaluate the time required to induce initial adaptive changes and will enable optimization of the training protocols for different muscle groups. Moreover, in this study only three external loads were used and it is possible that other external loads would greatly improve muscular strength and power. In addition, strength and power changes may be different when combined with other independent variables (set duration, set number, time of rest periods, etc). Toji and Kaneko (2004) found that multiple-load strength training was highly efficient. Therefore, in future studies we will apply a combination of different independent variables.

## Conclusions

The results of this study indicate that four weeks of ITMS training is effective in increasing shoulder joint adductors torque and power in physical education students, regardless of the external load used during training. The percentage changes in torque and power did not significantly differ between training groups. Moreover, using smaller loads (mass of the ITMS flywheel) results in neuromuscular coordination improvement. The findings of the present study indicate that training using a novel ITMS device is effective in increasing muscle strength and power. Accordingly, the application of inertial training for enhancing sport performance should be considered by coaches and athletes, although further studies are needed.

## Figures and Tables

**Picture 1:**
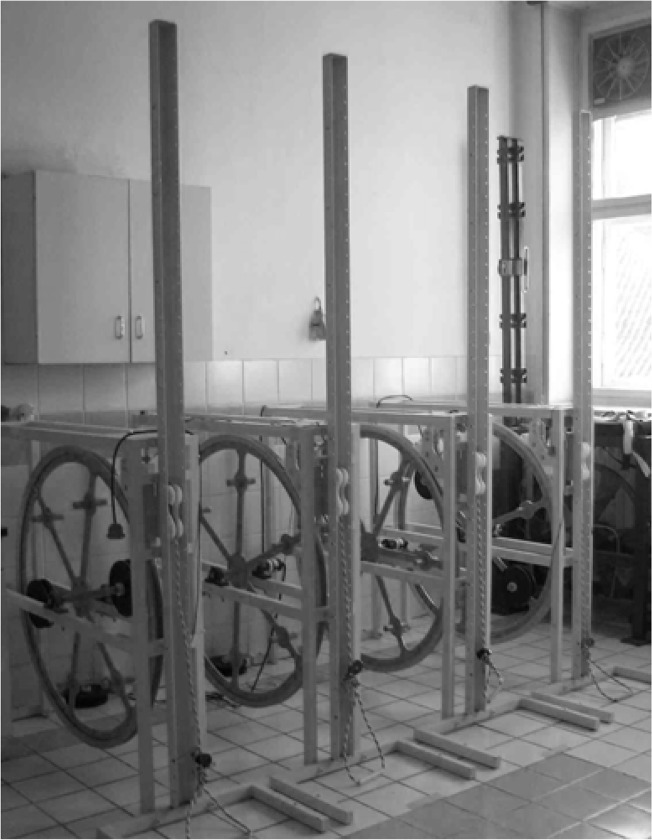
The Inertial Training and Measurement System

**Table 1:** Biometric characteristics of the participants

Group	n	Age [years]	Body height [cm]	Body mass [kg]
T0	11	20.4 ± 0.5	182.8 ± 7.8	74.2 ± 13.4
T5	11	20.9 ± 1.4	178.5 ± 6.5	73.0 ± 9.5
T10	11	21.0 ± 1.7	178.5 ± 6.5	76.2 ± 8.9
C	13	22.3 ± 2.1	177.2 ± 5.0	73.8 ± 10.4

T0 - group participated in training with no load in addition to the weight of the flywheel; T5- group participated in training with the flywheel loaded with additional 5 kg; T10 - group participated in training with the flywheel loaded with additional 10 kg; C - control group; results shown are the mean ± SD
